# Supplementary figures and images for: Gastric examination using a novel three-dimensional magnetically assisted capsule endoscope and a hand-held magnetic controller: A porcine model study
Source: PLoS One. 2021 Oct 5;16(10):e0256519. doi: 10.1371/journal.pone.0256519 (PMC8491884; doi:10.1371/journal.pone.0256519)

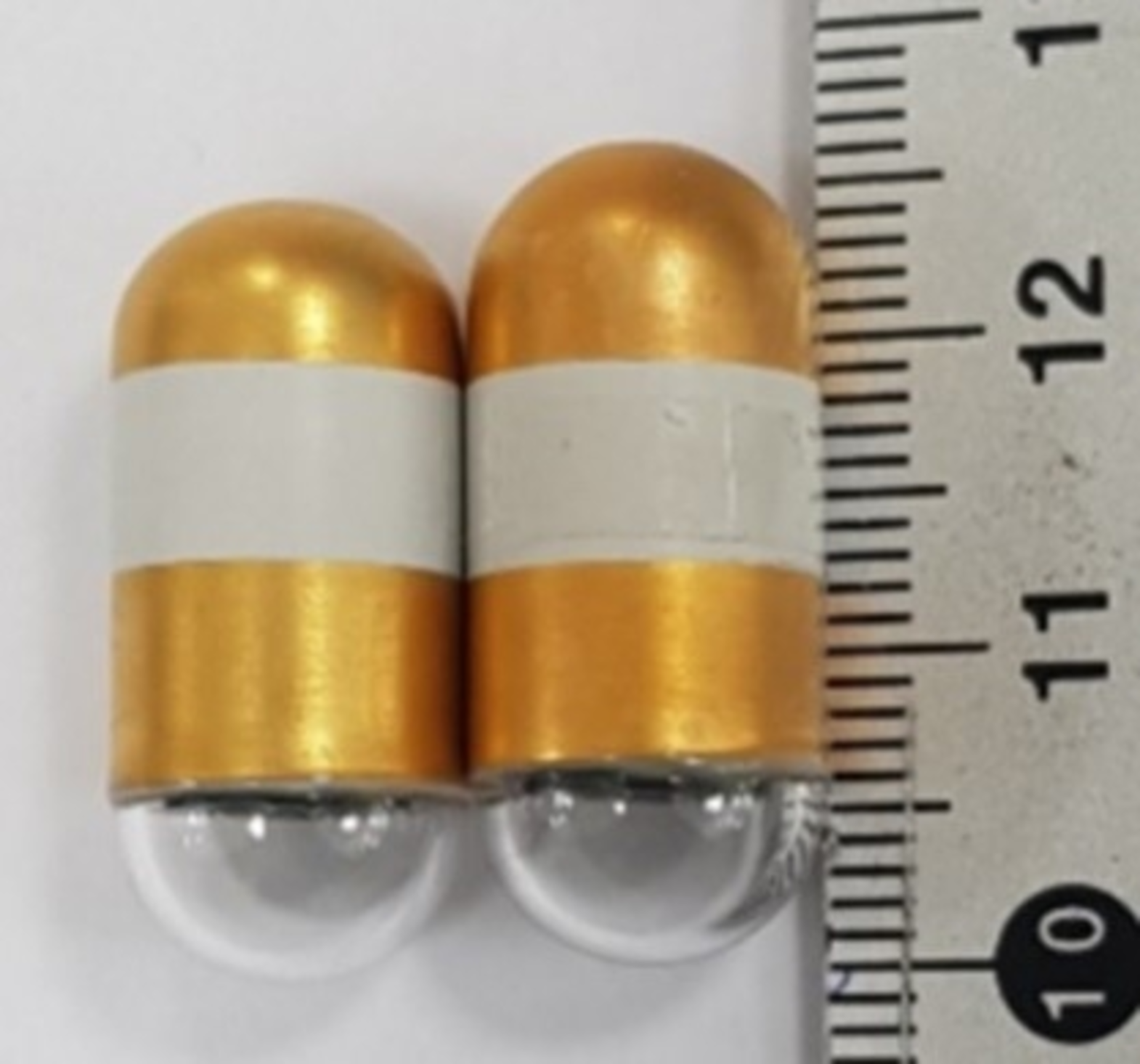

Supplement: S1 Fig — The left is conventional MACE, and the right is 3D MACE. 3D MACE is about 1mm longer than conventional MACE. (TIF) [file pone.0256519.s001.tif]

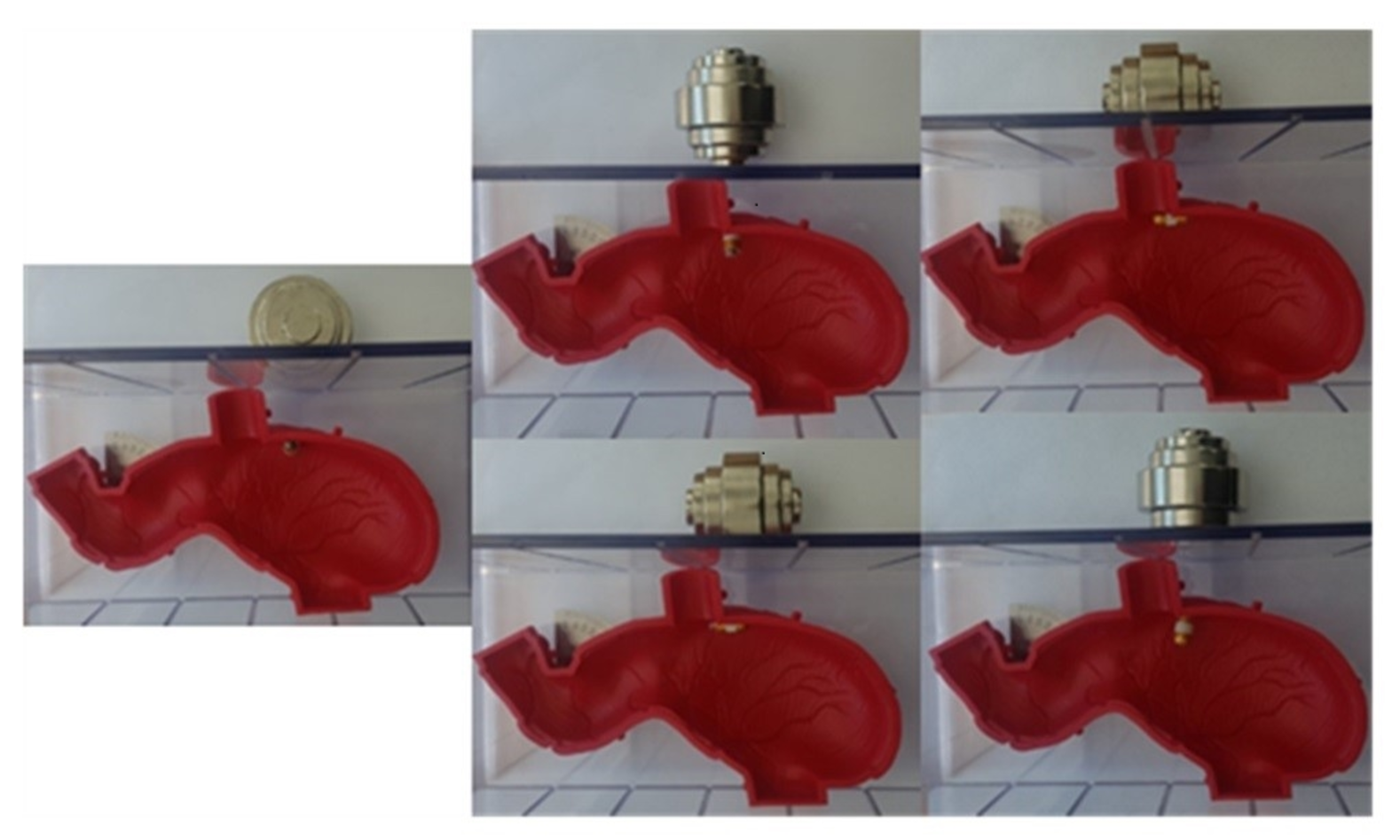

Supplement: S2 Fig — (TIF) [file pone.0256519.s002.tif]

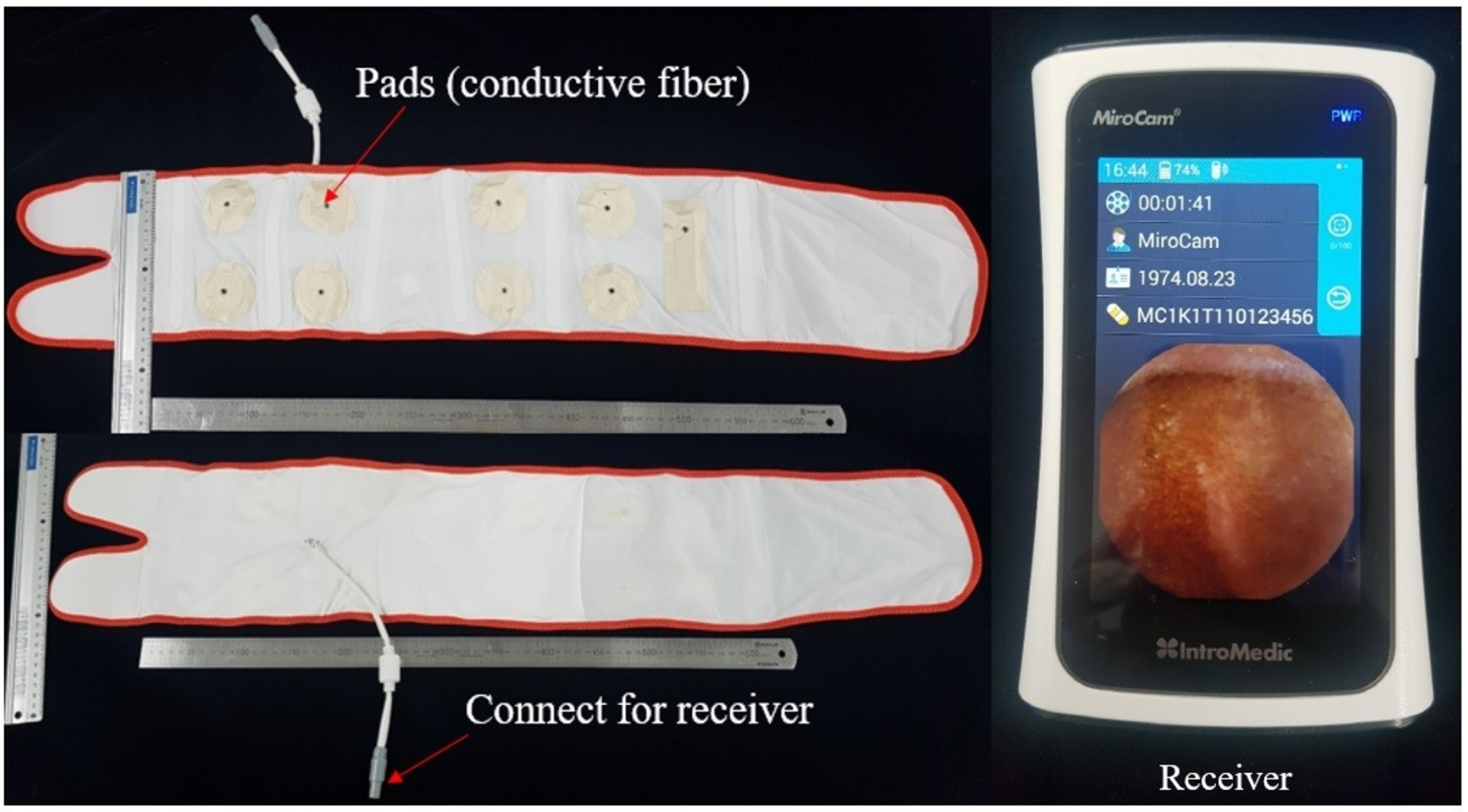

Supplement: S3 Fig — (TIF) [file pone.0256519.s003.tif]

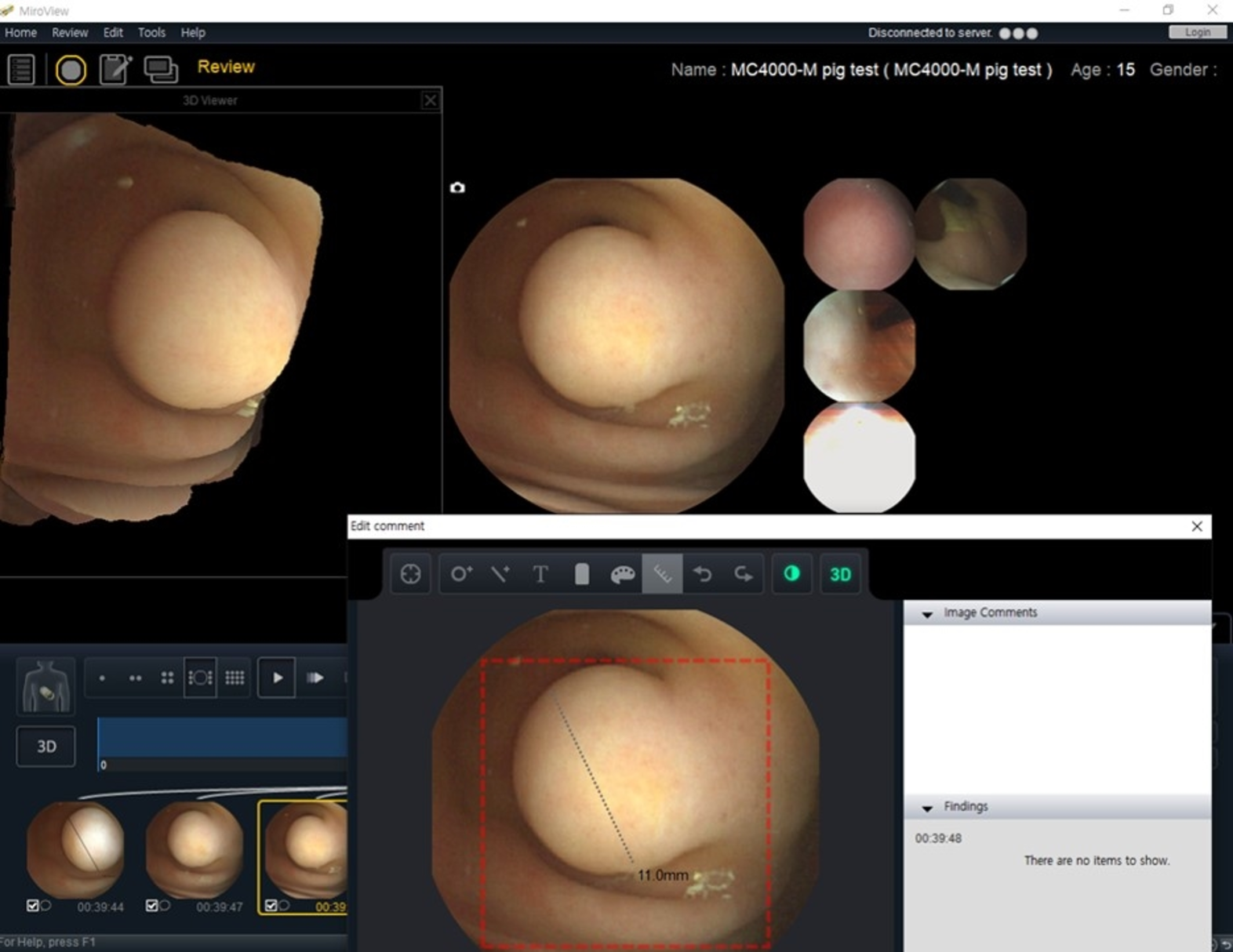

Supplement: S4 Fig — Three-dimension reconstruction of image and size measurement are possible with a new dedicated viewer. (TIF) [file pone.0256519.s004.tif]

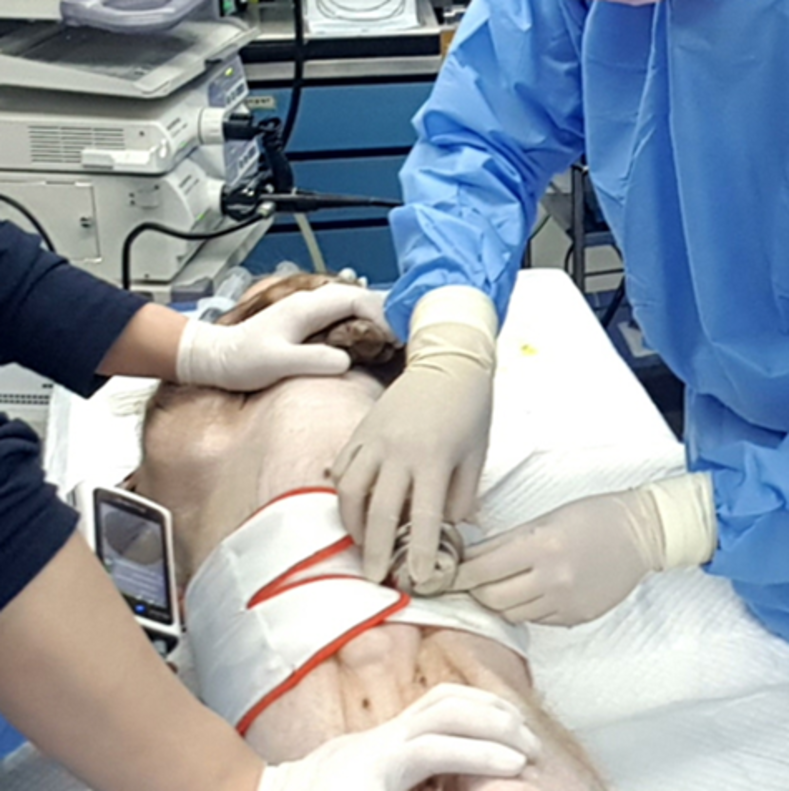

Supplement: S5 Fig — (TIF) [file pone.0256519.s005.tif]

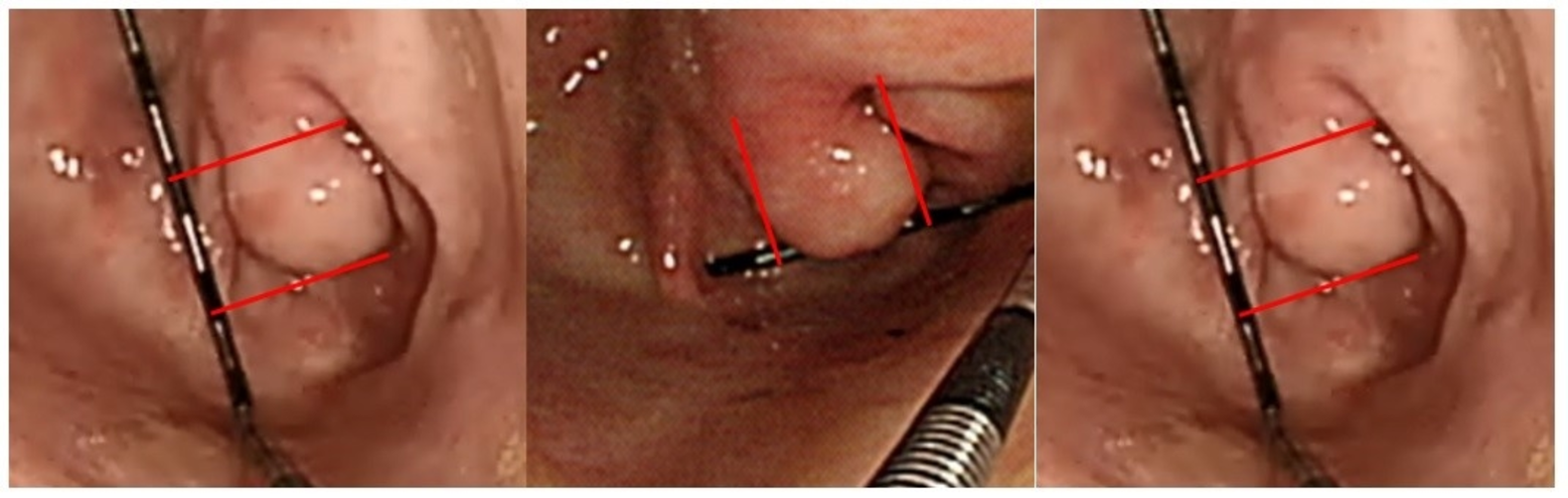

Supplement: S6 Fig — (TIF) [file pone.0256519.s006.tif]
