# Supplementary material for: Gastric examination using a novel three-dimensional magnetically assisted capsule endoscope and a hand-held magnetic controller: A porcine model study
Source: PLoS One. 2021 Oct 5;16(10):e0256519. doi: 10.1371/journal.pone.0256519 (PMC8491884; doi:10.1371/journal.pone.0256519)
Supplement: Attachment — Submitted filename: Response to Reviewers.docx [file pone.0256519.s008.docx]

First of all, thank you very much for giving the opportunity of revision. The authors appreciate the critical and thoughtful comments

We did our best to write the reply for your comments and to revise the manuscript stating clearly how the text has been changed. And, we added more detailed information and images in the manuscript.

We indicated precisely where changes have been made in the manuscript, by changing the font to words or sentences on a yellow background. Additional answers were written in **underlined with bold type**.

**[Author’s Answers to Reviewer #1]**

- INTRODUCTION –

1. Please quantify or provide a range in frames-per-sec on what the 'high' in "development of high frame rate of CE camera" refer to.

: **We added the sentence based on your comments.**

[Page 3, Introduction]

Along with the development of high frame rate of CE camera, some studies reported the use of CE for the evaluation of esophagus and colon (small bowel CE at 2-6 frame rate, esophagus and colon CE at 18-35 frame rate).

- METHODS –

1. This first paragraph describes that "Laboratory animal breeding and experiment were carried out in strict accordance with ethical guidelines and carried out at KNOTUS Co. Ltd (Incheon, Korea), a certified facility." What are the ethical guidelines of KNOTUS? Please attach the guidelines as supplementary information or link it to a publically (publicly) available URL

: **It seems that the sentence is somewhat ambiguous. This does not mean that there are guidelines for KNOTUS itself. It means that animal experiment has been carried out by an authorized facility that is registered with the Korea Food & Drug Administration and complies with Korean guideline**

**(http://www.cdc.go.kr/board.es?mid=a40606010000&bid=0041&act=view&list_no=123031)**

**: We revised the sentence based on your comments.**

[Page 4, Method, 1. Subject]

Laboratory animal breeding and experiment were carried out at animal experiment facility (KNOTUS Co. Ltd, Incheon, Korea) registered with Korea Food & Drug Administration.

2. Why was the study restricted to just one subject? Considering that the MACE was not able to pass the pylorus and travel through the small and large intestines because of the presence of the lesion, isn't this pre-clinical study preliminary and perhaps even incomplete to determine the efficacy of this 3D MACE and new magnetic controller?

**: First, it was difficult to pass through pylorus with a gastroscopy due to the lesion. Also, there will be differences in anatomy between human and mini pig (30kg). In humans, normal gut peristalsis alone allows capsule endoscope to pass through pylorus. Besides, in previous human studies using MACE, the capsule easily passed through pylorus by controller.**

**: Additional animal experiment using other controllers (the same magnetic force but kettlebell and trackball shape) was conducted in 2021 (KBIO-IACUC-2020-219). In this study, 3D MACE was passed through pylorus. We will attempt the complete stomach and lower GI tract examination through clinical study.**

3. Fig 1A, B, and C are generally of very low quality and appear not in focus. Please replace these images with higher quality images.

(a) - Please use a scale bar and mark the dimensions of the capsule in Fig1A.

**: We added the figure based on your comment in “S1 Fig”.**

(b) - The pictures of the capsule in Fig1A do not clearly show the 2 camera system that is important detail in this novel capsule. Please replace it with a macro-photograph that showcases the camera and imaging system on the capsule clearly.

**: We revised the figure based on your comment.**

(c) Fig C is also of very low quality. None of the text on display nor the label on the product is legible.

**: We revised the figure based on your comment. And we added the figure based on your comment in “S3 Fig”.**

4. The functionality of the real-time receiver and sensor belt has to be described. What sensors are used in the belt? What kind of receivers on the real-time receiver? Please include a schematic of the whole 3D capsular endoscopy system.

: **We added this information at “Method, 2. Devices” section.**

[Page 6, Method, 2. Device, (3) Real-time receiver with sensor belt]

Real time receiver uses Android version 9, with an LCD resolution of 800 x 480 pixels. The receiver and sensor belt can be connected. The pads of the sensor belt are composed of 9 conductive fibers (S3 Fig). The images recorded by the 3D MACE are delivered to the real time receiver using a novel transmission technology, known as electrical field propagation. This technology has been applied from the existing small bowel capsule endoscope of the Intromedic Co. Ltd [16].

5.

(a) In Section 2. Devices, Subsection (2) Hand-held magnetic controller, it is not clear what a conventional controller is? Is it an existing Intromedic product? What are its dimensions? How does the new magnetic controller compare to other research-stage or other commercial handheld controllers? Please provide references to other controllers as well. What would be most appropriate is if controllers and their magnet configurations can be compared in a figure.

: **The conventional and existing controller is a hammer shape only (not commercial). The hammer shape controller is 26 cm length. So, we studied new controllers (circular-stacked,) with easy-to-manipulate shapes and sizes. We revised the “Fig 1C” based on your comments**

[Page 6, Method, 2. Device, (2) Hand-held magnetic controller]

The new controller is smaller in size than an existing controller (hammer shape, 26 cm in length, 6.5cm in hammer head width) [11].

Figure 1. Overview of the three-dimensional (3D) magnetically assisted capsule endoscopy (MACE) system. A: 3D MACE (MiroCam® MC 4000-M), B: circular stacked magnetic controller, C: real-time receiver with sensor belt (MR 2000). The size of the existing controller and the new controller was compared.

(b) It is mentioned that "the magnetic force is equal between the two"? How has this been calculated or estimated. The other sections of the paper do not provide satisfactory explanation to this.

: **Although the size of the new controller is smaller than that of the hammer-shaped controller (7 cm versus 26 cm), it uses a high-grade magnet (N54 versus N35). So, the measured magnet force was similar in the new and existing controller.**

[Page 6, Method, 2. Device, (2) Hand-held magnetic controller]

But new controller is equipped with a higher-grade magnet than the magnet of existing controller (N54 versus N35). So, the magnetic force measured 10cm away from the controller was similar in the new and existing controller. (30 gauss in new controller and 27 gauss in existing one, respectively).

- RESULTS -

1. In Fig 4C, it’s very hard to visually distinguish a remarkable difference between conventional MACE and 3D MACE. Could another image of a different region be provided that shows a marked difference?

**: We revised the figure based on your comments.**

2. In the section on Safety,

(a) I note that the MACE was released from the pig only after 7 days post the experiment. Is the delay primarily due to the fact that the presence of the lesion did not allow the capsule to enter the duodenum?

: **We put the 3D MACE into the duodenum using the gastroscopic net. So, it took 7 days to release 3D MACE from the small intestine to the anus. Release of 3D MACE was confirmed by experimental facility. We added this information to the manuscript.**

[Page 11, Results, 3. Safety]

No significant adverse event was observed when reconfirmed by upper endoscopy. We moved the 3D MACE into the duodenum using the gastroscopic net. The chest radiograph also showed no abnormal findings. The 3D MACE was released from the mini-pig 7 days after the experiment, without any complications.

(b) How does the capsule staying within the pig for 7 days, affect the subject? Is there some part of the ethical guidelines of KNOTUS facility which deals with such a situation?

**: After experiment, the subject recovered without any complications and was observed in the experimental facility until the capsule was released. We also checked the breeding records in KNOTUS facility.**

- DISCUSSIONS -

1. Is the difference between MC4000 and MC4000-M, only the presence of the magnetic controller, or is there something else?

**: The MC4000-M is a new capsule that contains a magnetic inside the MC 4000. There are minor changes in weight and size (0.3 g and 1 mm), but there are no other differences.**

2. Regarding the hammer-shaped magnetic controller, please refer to my questions in the Methods section about comparing existing magnetic controllers. Ideally please include a comparative imagery of existing handheld magnetic controllers w.r.t to the new design?

**: We added the comparative figure at “Fig 1” based on your comments.**

3. "Therefore, the calculated magnetic forces of the two controllers are similar". The explanations given to explain the force equivalent is unsatisfactory, if dimensions are not provided of conventional controller nor analytical or magnetostatic simulations regarding this.

**: We measured magnetic force and added this information to the “Method” section.**

4. As I mentioned earlier, I do not believe one of the concluding statements "This preclinical trial provides a basis for further clinical studies on 3D MACE." since the submitted manuscript has not detailed a complete GI tract examination and the present study seems to be incomplete and needs further preclinical trials on multiple subjects before becoming a basis for clinical studies.

**: Of course, there is still a long way to go for complete GI tract examination. Among the several problems, the active locomotion of capsule endoscope has been solved through many studies. However, measuring the size of lesion and reconstructing stereoscopic images by capsule endoscopy have yet to be studied much. The goal of this study was to develop and verify wireless capsule endoscope that can measure the size of lesions and reconstruct conventional images into 3D images. We will try to solve the remaining problems through further researches.**

**[Author’s Answers to Reviewer #2]**

1. Furthermore, I would prefer a title like: Gastric examination using a novel three-dimensional (3D) magnetically assisted capsule endoscope and a hand-held magnetic controller: A porcine model study.

**: We revised the title based on your comments.**

2. Although the majority of the images are useful and of high quality, I believe that the main set up of the experiment, that is (if not a real image) please consider adding an infographic showing the set up of the experiment.

**: We added more detailed images in “S4 Fig”**
