# Supplementary material for: Gastric examination using a novel three-dimensional magnetically assisted capsule endoscope and a hand-held magnetic controller: A porcine model study
Source: PLoS One. 2021 Oct 5;16(10):e0256519. doi: 10.1371/journal.pone.0256519 (PMC8491884; doi:10.1371/journal.pone.0256519)
Supplement: Attachment — Submitted filename: Response to Reviewers.docx [file pone.0256519.s009.docx]

First of all, thank you very much for giving the opportunity of revision. The authors appreciate the critical and thoughtful comments

We did our best to write the reply for your comments and to revise the manuscript stating clearly how the text has been changed. And, we added more detailed information and images in the manuscript.

We indicated precisely where changes have been made in the manuscript. Additional answers were written in **underlined with bold type**.

**[Author’s Answers to Reviewer #3]**

- Methods 2. Devices (2) Hand-held magnetic controller –

1. Please specify the effective operation distance (in cm) between the controller and the 3D MACE.

: **We added the sentence based on your comments. The magnetic force measurement was conducted in the Department of Mechanical Engineering, Hanyang University.**

(The effective operation distance between the magnetic controller and 3D MACE is up to 12.5cm.)

2. The authors only mentioned the magnetic flux density (in Gauss) 10 cm away, but not the corresponding magnetic force (in Newton) with respect to the MACE. Please complement this information.

**: We complemented this information. The magnetic force measurement was conducted in the Department of Mechanical Engineering, Hanyang University**

(The magnetic force with respect to the MACE by the controller was measured to be 41.2 mN at a distance of 10.5 cm.)

3. If during the operation, the hand-held controller is accidentally moved beyond the effective range with the MACE, would it lost the pose control of MACE? If this is the case, how to re-localize MACE and restore control of MACE? Please comment.

**: There were no "significant lost control" studies during two additional porcine studies. Even if it was out of effective range, it was not difficult to re-localize the MACE by moving the controller using the real-time viewer.**

- Methods 3. Porcine gastric examination... –

Please add a picture or a general schematic of the experiment setup, including the test subject (i.e. the mini-pig) together with the arrangement of all the devices.

**: We added actual picture of animal experiment in Supplementary material (S6 Fig)**

- Results 2. Verification of images of the gastric... -

The result in terms of lesion size estimation is not quantitatively sound. Eventually only one set of data was reported and assessed. It would be recommended to perform additional test-bench characterization to quantitatively assess the accuracy of the lesion size estimation.

**: We conducted a study on object size estimation using Korea Electronics Technology Institute (KETI) test program before animal experiment. In a human intestinal model, polyp models of various sizes were randomly placed. Then, when the size measurement was performed using MACE, the accuracy of 93.37% was confirmed.**

- Supplementary material -

In S2 Fig, different orientations of MACE were shown, however, it would be really interesting to see a supplementary video of the pose (position and orientation) control of MACE under the guidance of the hand-held controller.

**: We added a short video confirming the movement of MACE under gastroscopy (S5 Video).**
